# Unusual Defect Chemistry of Thorium Doping of PbS

**DOI:** 10.1021/acs.inorgchem.4c02998

**Published:** 2024-10-13

**Authors:** Neeraj Mishra, Shachar Moskovich, Michael Shandalov, Eyal Yahel, Yuval Golan, Guy Makov

**Affiliations:** † Department of Materials Engineering, Ben-Gurion University of the Negev, Beer-Sheva 8410501, Israel; ‡ Ilse Katz Institute for Nanoscale Science and Technology, Ben-Gurion University of the Negev, Beer-Sheva 8410501, Israel; § Department of Physics, Nuclear Research Center Negev, Beer Sheva 84190, Israel

## Abstract

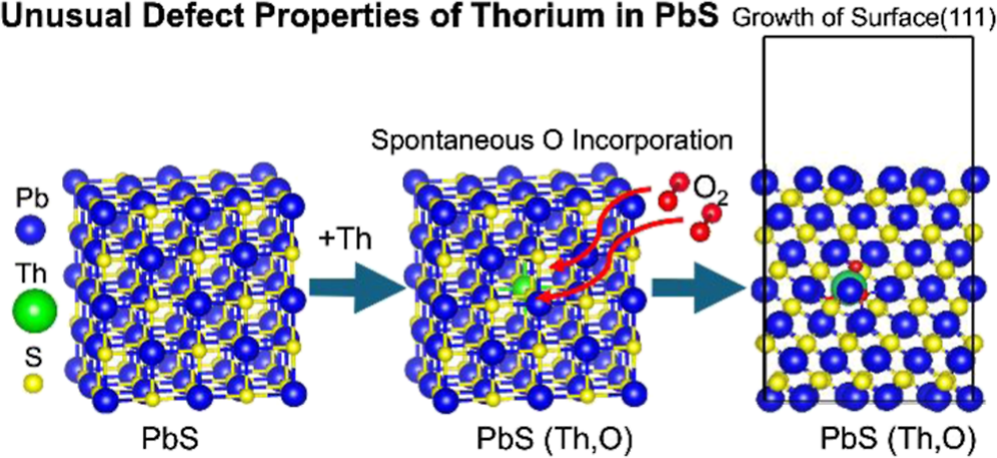

The unusual defect chemistry of thorium doping in the PbS system was investigated computationally to answer several open questions arising from the experimental observations. These include finding Th in a +4 oxidation state in contrast to Pb, attracting more than two oxygen atoms on average per thorium and affecting the growth morphology of PbS and its electronic properties. We find Th to be energetically stable at the lead lattice position in PbS and to attract 2–3 oxygens, including in the adjacent interstitial position, which binds strongly to Th. This adjacent interstitial atom allows the +4 oxidation state of Th in PbS as observed experimentally. Furthermore, the bandgap of the ideal material increased due to Th incorporation, in agreement with experimental observations. Finally, we calculated the surface energies of the (100), (110), and (111) surfaces for the systems with and without thorium incorporation. Surfaces (100) and (110) were found to have negative surface energies; however, (111) surface energy was positive and, thus, preferred for the growth of Th-doped PbS thin films. These results correlate well with the experimentally observed surface topography change for PbS thin film growth from the (100) to the (111) surfaces with addition of Th.

## Abstract 2

The observed unusual defect properties of Th in PbS, including enhanced oxygen absorption and selective growth, are studied by density functional modeling. We find that Th assists in the incorporation of multiple oxygen atoms into specific sites of the crystal lattice. We model possible surfaces of defect-incorporated PbS and find that only the (111) surface is stable, indicating preferred growth in this direction. Our results agree very well with the experimental observations.

## 1 Introduction

Lead sulfide (PbS), the most studied material among the group IV monochalcogenides, is a narrow, direct band gap semiconductor.^1^ The interest in PbS is due to its optoelectronic properties, useful for infrared detection and emission.^2−4^ Recently, interest in nanocrystalline PbS has increased significantly due to potential applications in solar cells and visible light sensors.^5−7^

Doping is a well-known and effective technique to modify the structural, optical, and electrical properties of semiconductor materials. In PbS, doping of several elements has been reported, which can alter the resistivity, optical bandgap, and carrier density.^8,9^ Researchers have studied multiple dopants, such as Ag, Al, Cu, Cd, Zn, Sb, and Sn, to tailor the optical and electrical properties of PbS.^8−22^ An increase in the optical bandgap and decreased electrical resistivity were obtained with Al doping in PbS,^11^ whereas Ca and Sn dopants decrease the bulk band gap of PbS.^20,22^ Introducing Ag and Cu dopants in PbS thin films increased the carrier concentration and decreased resistivity.^21,22^ The thickness of the thin film was found to decrease with increasing Sn doping,^20^ which XPS analysis indicates to be in the +2 oxidation state.^20^ Touati et al. reported a modification in the as-deposited surface morphology of Ag-doped PbS thin films deposited on a glass substrate. Surface texture changed to (200) from (111) for doping concentrations in the range of 2–4%.^21^

Thorium (Th) ions are an alternative dopant of PbS that is tetravalent and could be expected to show unique effects. Indeed, Th doping of PbS has very recently been studied by some of the authors,^23−26^ where their initial motivation was the investigation of radiation damage using the ^228^Th isotope in PbS thin films.^27^ Biton et al. deposited ^232^Th-alloyed PbS thin films at various concentrations on GaAs substrates using chemical solution deposition.^23^ They found that low concentrations of Th (0.5%) strongly affected the surface topography and slowed the growth rate of the films.^23^ Addition of thorium changed the surface topography of the films from a faceted rectangular morphology (indicative of the [110] orientation) to a triangular morphology (indicative of the [111] orientation).^23^ They suggested that these changes in the film texture to <111> could be due to Th adsorption on the GaAs substrate.^23^ Templeman et al. studied the effect of increased thorium content in PbS thin films.^24^ They found that Th^4+^ acts as a growth inhibitor and reported that thorium alloying reduces film thickness under the same deposition conditions.^24^

Thorium alloying also strongly affected the structural, chemical, and electronic properties of PbS.^23,24^ Th ions were incorporated in chemically deposited PbS thin films and were found to be tetravalent.^23,24^ Chemical analysis of film composition and dopant distribution was performed by XPS depth profiling. Th^4+^ and O^2–^ ions, in addition to Pb and S ions, were uniformly distributed throughout the film. Surprisingly, substantial oxygen content was found in Th-doped PbS thin films, up to concentrations of 9 at % thorium and 20 at % oxygen, with a typical O/Th ratio of 2.4.^24^ A charge compensation mechanism for the local incorporation of oxygen and thorium in PbS(Th, O) was suggested.^24,25^

Furthermore, the electronic properties of thorium-alloyed PbS thin films were affected by optical measurements, indicating a blue shift in the energy bandgap toward the short wavelength infrared range (SWIR).^24,25^

These experimental observations open several questions regarding the defect chemistry of Th in PbS. Specifically, (i) why does oxygen accumulate in large numbers in the presence of Th? (ii) What are the preferred locations of the Th and oxygen atoms in PbS? (iii) What is the cause of the increase in the observed band gap, and (iv) why does the preferred orientation in thin film growth change upon introduction of Th? The present study of thorium dopants in PbS applies first-principles modeling to study their chemistry and effect on physical and material properties. We also explore the surface energies and bandgap of thorium-doped PbS and find our theoretical results to be in good agreement with experimental reports, providing answers to the questions raised.

## 2 Methods

Formation energies of intrinsic and extrinsic point defects (dopants) in rock-salt PbS (space group *Fm*3̅*m*) were calculated in supercells with periodic boundary conditions using density functional theory (DFT). All calculations were performed using the first principles DFT package Quantum ESPRESSO,^28^ with projector augmented wave (PAW) methods. The PBEsol variant of the Perdew–Burke–Ernzerhof (PBE) generalized gradient approximation^29^ was employed for the exchange–correlation energy with scalar relativistic corrections (nonlinear core corrections included) to determine lattice constants and formation energies. This method predicted the PbS bandgap in reasonably good agreement with the experiments. We found the bandgap of the ideal material to be 0.35 eV and in good accord with the experimentally reported value of 0.30 eV at lower temperatures.^30^ The optimized lattice parameters of the PbS unit cells were computationally determined. The cutoff energy of the plane-wave expansion of the wave functions was 80 Ry. A supercell of 3 × 3 × 3 multiples of rock salt’s two-atom face-centered cubic (FCC) primitive cell was used. The *k*-point sampling employed in calculations was 4 × 4 × 4 for 54-atom supercells. Unit cell calculations were performed with a 16 × 16 × 16 *k*-point mesh. We performed convergence tests of the final total energy with respect to the energy cutoff and *k*-point mesh sizes. The convergence tolerance for forces on each ion is less than 0.01 eV/Å, resulting in the convergence of the total energy to less than 10^–5^ eV.

Elemental phase energies were calculated for molecular oxygen, FCC lead and thorium, and orthorhombic α-sulfur, all of which are necessary to calculate defect formation energies. Elemental calculations for sulfur and oxygen employed 4 × 4 × 4 and 1 × 1 × 1 *k*-point meshes, respectively.

The formation energy is central to determining the relative thermodynamic stability of point defects and can be determined from^31^
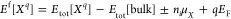
1where *E*^f^[*X*^*q*^] is the formation energy of defect *X* with charge *q*, *E*_tot_[*X*^*q*^] is the total energy of a supercell containing a defect after the relaxation of the ion positions, *E*_tot_[bulk] is the total energy of a bulk (perfect) supercell with the same number of atoms, *n*_*i*_ is the number of atoms added and removed from the supercell, μ_*X*_ is the chemical potential of atoms, and *E*_F_ is the Fermi energy. For a neutral defect, *q* equals zero in eq 1.

In modeling charged defects in insulators and semiconductors, the Fermi energy (in the last term of eq 1) is assumed to vary between the valence band maximum (VBM) and the conduction band minimum (CBM) depending on the nature of the semiconductor (n- or p-type) and the external electric potential. The PbS system is intrinsically p-type; hence, we compared the formation energy of charged defects with the Fermi energy at the VBM.

The chemical potentials are required to determine the defect formation energies. By convention, chemical potentials are referenced to their elemental bulk phases. Bulk (elemental) phase energies were calculated for molecular oxygen, face-centered cubic (FCC) thorium, lead, and orthorhombic α-sulfur. However, the allowed values of the chemical potentials of each component (Pb and S) in PbS follow
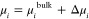
2

The allowed values of Δμ_*i*_ are governed by the above eq 2. To maintain a stable PbS compound, the chemical potentials of Pb and S must satisfy the equation μ_Pb_ + μ_S_ = *H*^f^ (PbS) + μ_Pb_^bulk^ + μ_S_^bulk^, or equivalently, Δμ_Pb_ + Δμ_S_ = *H*^f^ (PbS), where *H*^f^ (PbS) is the heat of formation of PbS. Moreover, chemical potential values of Th and O are restricted, so formation phases (e.g., ThS_2_ and PbO) of dopants with host components can be prevented, and PbS remains stable after doping. All of the defect formation energies were calculated under S-rich conditions in order to be consistent with the experimental conditions.

## 3 Results and Discussion

The lattice parameter of PbS in the rock salt phase and its formation energy were calculated to be 5.981 Å and 1.08 eV, respectively. These results can be compared with the experimentally determined values of 5.940 Å^32^ and 1.02 eV ± 0.05^32^ and are in excellent agreement with our previous theoretical values of 5.981 Å and 1.1 eV using other calculation methods.^33^ Thorium-substituted defects were modeled in PbS, and their detailed defect chemistry with oxygen was studied. We performed a convergence test for the defect formation energy of selected defects with supercell size to avoid the unphysical interactions of defects with their periodic images. Forces on each atom in the defect-containing system after relaxation were found to be less than 0.025 eV/Å. The formation energies of the defects were found to converge to better than 0.25 eV for Th and 0.01 eV for O for our choice of supercell size (3 × 3 × 3). Correspondingly, we observed that the incorporation of Th did not introduce significant strain into the PbS lattice, with the strain of the supercell remaining below 1%.

### 3.1 Thorium in PbS

Defect formation energy is the central quantity in the thermodynamics of defects, and formation energies were calculated for Th in PbS. The formation energies of substitutional Th defects (Th_Pb_) were found to be negative, indicating an easy formation of these defects in PbS (see Table 1). In contrast, a relatively large and positive formation energy was found for Th in the interstitial site (*I*_Th_). Therefore, we precluded further consideration of the interstitial configurations of Th in our study.

**Table 1:** Formation Energy (eV) for Thorium in PbS in the Presence and Absence of Oxygen

types of defects	formation energy (eV)	types of defects	formation energy (eV)
Th_Pb_	–0.18	*I*_Th_	3.25
*I*_O_	1.27	Th_Pb_ + *O*_S_	–0.65
*O*_S_/2*O*_S_	0.52:1.02	Th_Pb_ + 2*O*_S_	–0.61
Th_Pb_ + *I*_O_	–2.13	Th_Pb_ + *I*_O_ + *O*_S_	–2.73
Th_Pb_ + 2*I*_O_	–0.91		

Formation energies for Th with and without neighboring oxygen atoms are listed in Table 1. These results manifest that introducing O into the system further stabilizes Th-alloyed PbS.

From the results presented in Table 1, O atoms prefer to occupy substitutional sites (*O*_S_) over interstitial sites (*I*_O_) in bulk PbS. In contrast, in the presence of a Th atom, O prefers to occupy the adjacent interstitial site. Furthermore, this diatomic defect has a lower formation energy than Th-substitution, and the interstitial oxygen is bound to Th with a binding energy of 1.8 eV. It also manifests a +4 oxidation state of Th. It should be noted that Th substituting Pb in PbS is in a +2 oxidation state; however, interstitial oxygen (*I*_*O*_) acts as an oxidizing agent for two additional electrons of Th and oxidizes it to a +4 state. These results align with the reported observations.^24^

Moreover, we found that the formation energies were reduced when further oxygens were incorporated into the system. Therefore, thorium attracts oxygen into the PbS lattice, in good accord with the experiments.

### 3.2 Role of Oxygen

Two aspects were explored to further understand the role of oxygen in the defect chemistry of Th in PbS: (a) accumulation of oxygen in PbS in the presence of Th and (b) charge states of interstitial oxygen atoms.

We investigated the number of oxygen atoms that accumulated around a thorium atom by calculating the formation energies of Th atoms with increasing numbers of oxygen atoms around them.

**Figure 1:**
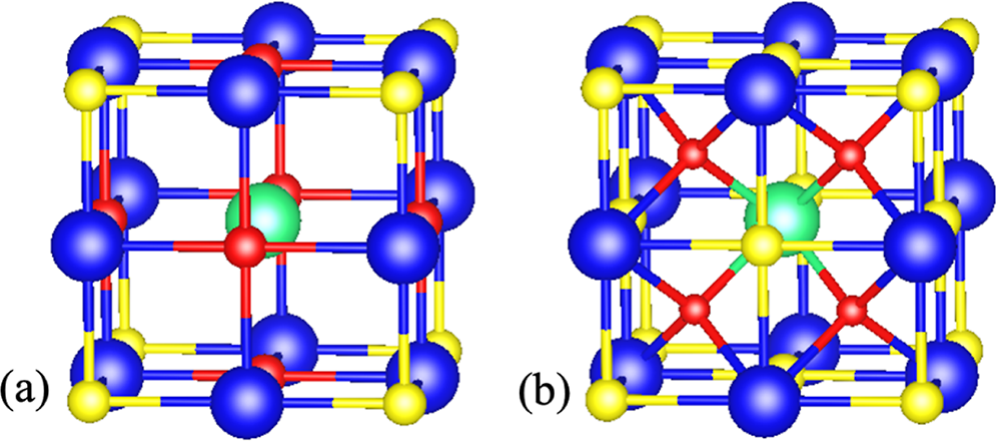
Schematics of nearest sites for O occupancy in defect configurations in Th-incorporated PbS: (a) substitutional and (b) interstitial. Blue, yellow, green, and red atoms represent lead, sulfur, thorium, and oxygen, respectively.

Specifically, we examined the substitutional and interstitial sites occupied by oxygens in various configurations to obtain their preferred arrangement. The defect configurations of oxygens in the Pb(Th)S system are represented by the chemical formula of the supercell
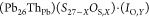
awhere Th_Pb_ and *O*_S_ are the substitutional defects and *I*_O_ represents the interstitial oxygen. *X* and *Y* are integers. The concentration of Th doping in our study was fixed at 3.7 *a*/0. The concentration of the O impurity can vary in the system with respect to the occupancy at substitutional and interstitial sites (see Figure 1). *X* and *Y* determine the occupancies of substitutional and interstitial sites in the system using a.

#### 3.2.1 Accumulation of Oxygen Atoms

The formation energies for the selected configurations are reported in Table 2. The rows (periods) in Table 2 represent increasing numbers of oxygen atoms (*X*) substituting sulfur at constant interstitial occupation (*Y*). For *X* = 1 to 6, O substituted nearest neighboring sulfurs due to the 6-fold coordination; however, for *X* > 6, the second nearest sulfurs are also substituted. Similarly, columns (groups) represent an increasing number of interstitial oxygens present at a fixed substitutional occupancy.

**Table 2:**
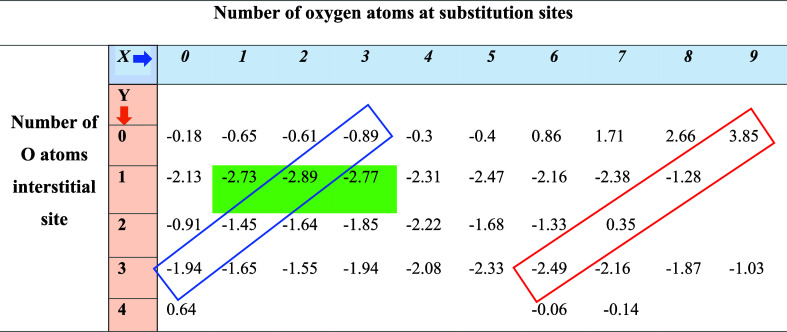
Formation Energies (eV) for Several Configurations with Oxygens at Substitutional and Interstitial Sites^a^

a The stable window of configurations is highlighted in green. The blue and red diagonally oriented boxes represent configurations with the same total number of oxygen atoms.

To understand the defect chemistry, we consider the stepwise addition of O atoms in the system. In the absence of an interstitial O, the formation energy was found to decrease (become more negative) with an increase in the number of oxygen atoms up to two and then increase. The results in Table 2 show that the system gains significant stability when an O occupies the interstitial site followed by the substitutional site. For instance, we consider a hypothetical system in which a single O atom is available, and occupancy of the interstitial site leads to a significant gain of energy (−2.13 eV), greater than that for the substitutional site (−0.65 eV), due to the formation of the stable oxidation state of thorium +4. Adding a second interstitial oxygen leads to a smaller gain in energy (−0.91 eV), whereas significant energy is gained (but less than for the single interstitial) (−1.94 eV) for the third O introduced at an interstitial site (see Table 2). However, the system requires significant energy (0.64 eV) to incorporate a fourth oxygen atom at the interstitial site.

**Figure 2:**
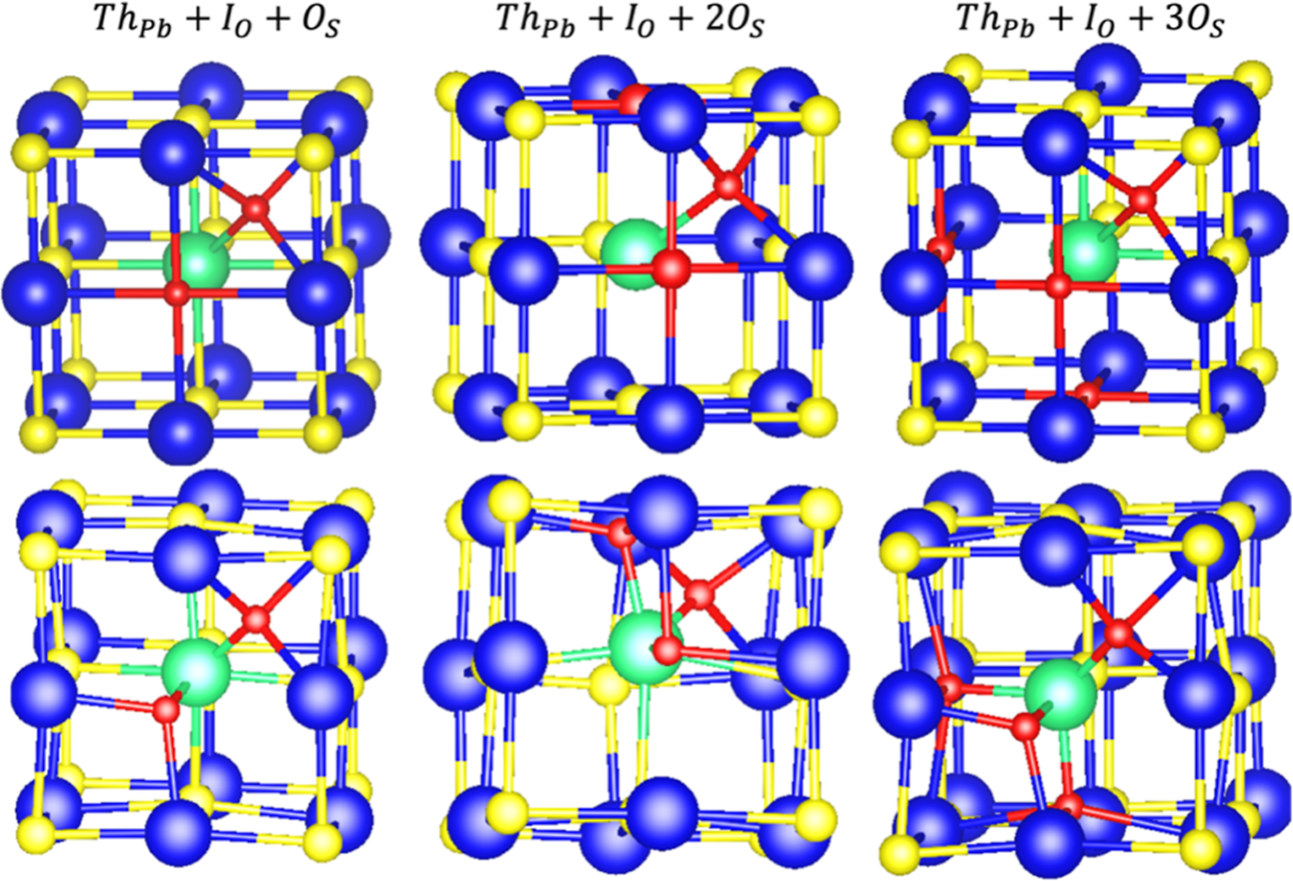
Initial (first row) and optimized (second row) geometries of the most stable PbS(Th, O) defects, representing part of the computational supercell. Blue, yellow, green, and red atoms represent lead, sulfur, thorium, and oxygen, respectively.

From the results presented in Table 2, we found a stability window of the most energetically stable configurations; each stable configuration has an O atom at the interstitial site and 1–3 substitutional oxygens. Their structural schematics are shown in Figure 2 and exhibit a small localized distortion near the location of the interstitial oxygen. Furthermore, we compared the values of the formation energies across diagonals in Table 2, for which the total number (interstitial and substitutional) of oxygen atoms is equal. Configurations with one oxygen atom at the interstitial site presented an energetically stable arrangement among the other possible configurations for an equal number of oxygen atoms from *X* = 1 to 7. However, configurations in which three oxygen atoms occupy interstitial sites become relatively more stable for further increase in *X*, reflecting the relative attractivity of interstitial oxygen sites compared to second nearest neighbor locations. Nevertheless, the most energetically stable configuration was obtained for two oxygens (*X* = 2) at the substitutional sites and a single O (*Y* = 1) at the interstitial site.

**Figure 3:**
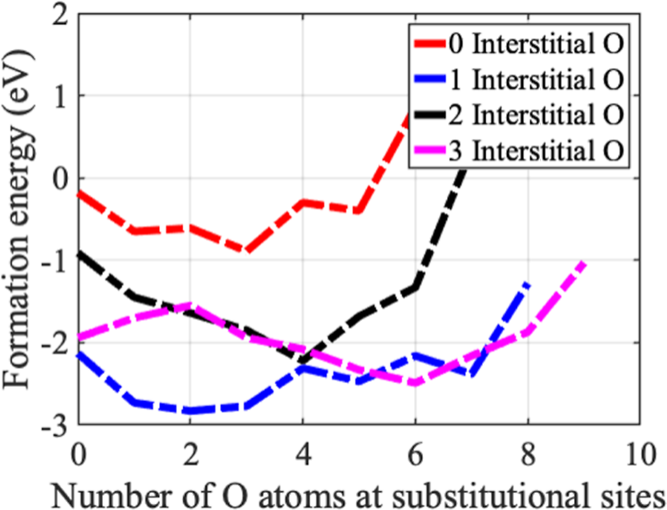
Formation energy (eV) of a Th substitution and a fixed interstitial O atom(s) with O atoms present at substitutional sites.

An alternative representation of the formation energies of Th–O defects reported in Table 2 is presented in Figure 3. The lowest formation energy among all Th–O configurations in PbS is for one O at the interstitial site (*Y* = 1) and two O at the substitutional sites (*X* = *2*). Therefore, we found that the average number of oxygen atoms accumulated in the Th-incorporated PbS system is three (one interstitial and two substitutional). Our results are in excellent agreement with the experimental reported results, which reported an average of 2.4 ± 0.1.^24^ Notably, a positive formation energy of the impurities of the O in pristine PbS indicated the endothermic process for their incorporation in the lattice of PbS (see Table 1). Hence, in contrast to Th-incorporated PbS systems, pristine PbS does not accumulate O in the lattice.

#### 3.2.2 Oxidation States of Thorium and Oxygen

Incorporating a large number of oxygens with Th in PbS may lead to the accumulation of negative charges in the system. Therefore, we investigated the charge stability of oxygen and Th in the system. Charge stability was calculated in selected defects, and the results are reported in Table 3. In a bulk system, only neutral charge states can exist. The theoretical charge state (C.S.) results should be correlated with the experimental oxidation states (O.S.). E.g., a neutral Th_Pb_ defect (C.S. *q* = 0) possesses the same oxidation state as Pb atoms in a bulk PbS system, i.e., +2. Accordingly, the computational charge state *q* = +2 is equivalent to the +4 oxidation state of Th.

**Table 3:** Charge State Stability Calculations of Interstitial O Defects in the Th-Incorporated PbS System^a^

types of defects	formation energy (eV)	types of defects	formation energy (eV)
Th_Pb_		Th_Pb_ + *I*_O_	
*q* = 0	2.17	*q* = 0	0
*q* = +2	0	*q* = +2	0.26
*q* = +4	0.88	*q* = +4	0.63
Th_Pb_ + 3*O*_S_		Th_Pb_ + 2*I*_O_	
*q* = 0	1.39	*q* = −2	0
*q* = +2	0	*q* = 0	0.45
q = +4	0.9	*q* = +2	0.7
Th_Pb_ + *O*_S_ + *I*_O_		Th_Pb_ + 2*O*_S_ + *I*_O_	
*q* = −2	2.1	*q* = −2	1.82
*q* = 0	0	*q* = 0	0
*q* = +2	0.25	*q* = +2	0.50

a Formation energies (eV) are calculated at VBM, which is taken as a reference. The stable configuration is set to zero eV.

First, we calculated the formation energies of the Th_Pb_ defect at the C.S. (*q* = −2, 0, and +2) and found that the lowest formation energy corresponded to the unphysical *q* = +2, which implies a +4 oxidation state. Next, we introduced O at the substitutional site along with Th, and a similar result was obtained, where the stable charge state (*q* = +2) corresponded to +4 O.S. of Th. In contrast, placing the oxygen in an interstitial position adjacent to the Th atom determines that the lowest energy state is neutral (*q* = 0). Thus, Th_Pb_ + *I*_O_ presents a +4 oxidation state of Th as extra two electrons of Th are accommodated by the interstitial oxygen, resulting in stable O.S. for Th and O of +4 and −2, respectively. Adding another interstitial oxygen requires the system to have a charge *q* = −2. In contrast, adding additional substitutional oxygens to a single interstitial oxygen allows the system to remain neutral. Therefore, we found that thorium is stable at a +4 oxidation state, while interstitial and substitutional oxygens are at −2 for all the defects, thus confirming the physical validity of the defect configurations identified in the stability window above. These results are in good accord with the experiments, where XPS depth profiling performed chemical analysis of Th-alloyed PbS film composition and distribution.^24^ A uniform distribution of Th^4+^ and O^2–^ ions was observed throughout the film depth.^24^ Hence, thorium, a tetravalent dopant, is stable in a +4 oxidation state in PbS.

### 3.3 Band Gap Variation Due to Thorium Defects

Point defects can alter the electronic structures of bulk materials. The band gaps of PbS with defects were calculated and are shown in Table 4. We found that the calculated band gap of ideal PbS (0.35 eV) is in good agreement with the experimentally reported value of 0.3 eV at lower temperatures.^30^ The introduction of thorium defects affected the material’s band gap significantly. We calculated the band gap for several selected point defects to understand the effect of Th and O introduction on the band gap of PbS. The material’s band gap was found to be significantly increased due to the presence of thorium defects for all defects considered (see Table 4). We found that the bandgap size of the Th_Pb_ + *I*_O_ defect is reduced compared to other Th-incorporated defects. This difference is due to the transfer of two electrons from Th to interstitial O, resulting in Th achieving a +4 oxidation state. We calculated the band gap of PbS for increasing Th concentrations and found it to increase with the thorium concentration in the system. However, we found a nearly unchanged band gap of PbS due to O substitutional defects, consistent with our previous work.^33^ These results emphasize that introducing Th increases the bandgap of PbS irrespective of the pH of the environments (for sample preparation). Moreover, the optical properties of Th^4+^- and O^2–^-incorporated films were studied experimentally to study the change in the physical properties of bulk PbS.^24^ The bandgap was reported to slightly increase with a low concentration of Th, whereas there was a significant increase in bandgap for Th concentrations above 7 at % in PbS.^24^ Biton et al. obtained similar results using PL measurements, and a small concentration of thorium increased the bandgap of PbS samples.^23^ Moreover, Arad-Vosk et al. measured bandgaps of the PbS(Th, O)-based photodiode, and a blue shift in the bandgap was obtained in optical characterization by introducing Th in PbS films.^25^ Therefore, our theoretical results for the band gap align well with the experiments.^23−25^

**Table 4:** Band Gap (eV) Response of Pristine PbS and with Thorium and Oxygen Defects

types of defects	band gap (eV)	defects	band gap (eV)
ideal supercell	0.35	Th_Pb_ + *O*_S_	0.58
Th_Pb_	0.58	Th_Pb_ + 3*O*_S_	0.63
2Th_Pb_	0.79	Th_Pb_ + 4*O*_S_	0.58
Th_Pb_ + *I*_O_	0.43	Th_Pb_ + 2*O*_S_ + *I*_O_	0.58
*O*_S_	0.33		

### 3.4 Surface Energies of PbS Surfaces

**Figure 4:**
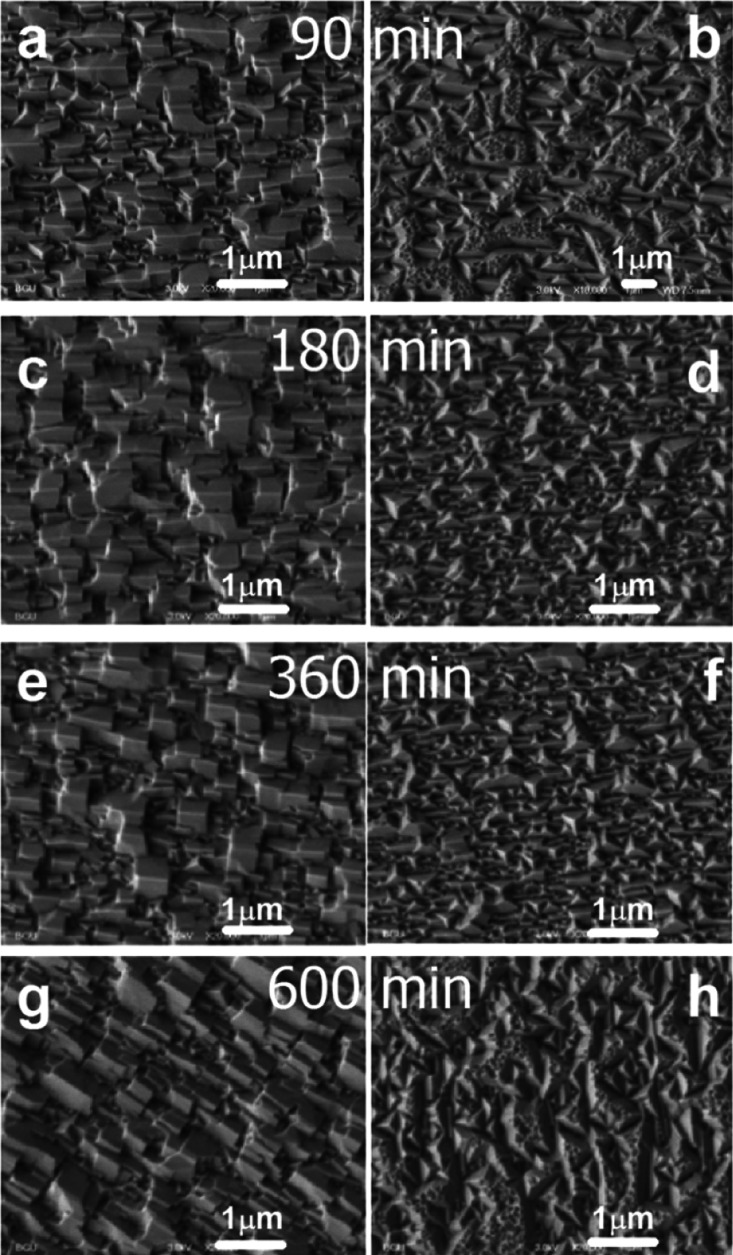
Effect of deposition time. Secondary electron HR-SEM images of (a,c,e,g) PbS thin films (without thorium); (b,d,f,h) PbS(Th) thin films prepared with a 0.438 mM thorium concentration in the solution and deposited on GaAs(100) at 30 °C for 90, 180, 360, and 600 min [published with permission from Elsevier, ref (23)].

Experimentally, introducing the thorium (tetravalent) dopant in small concentrations (0.5%) in PbS changed the surface morphology of the as-deposited PbS thin film (Figure 2). Surface topography of the as-deposited PbS film changed from (100) on the GaAs (110) substrate to (111) upon introducing the thorium dopant.^23^Figure 4 compares PbS and PbS(Th) films deposited at various deposition times under the same growth conditions (30 °C, 90–600 min). High-resolution scanning electron microscopy (HR-SEM) images in the plane-view reveal noticeable differences between chemically deposited PbS films with the addition of thorium and those without. The inclusion of thorium consistently led to a transition from [110] textured films to [111] textured films (see XRD in figure 8 of ref (23)). This transition is clearly manifested in the surface topography of the films, shifting from a faceted rectangle pyramid morphology characteristic of [110] textured films to a triangular pyramid morphology indicative of [111] textured PbS films, as seen in Figure 4.

**Figure 5:**
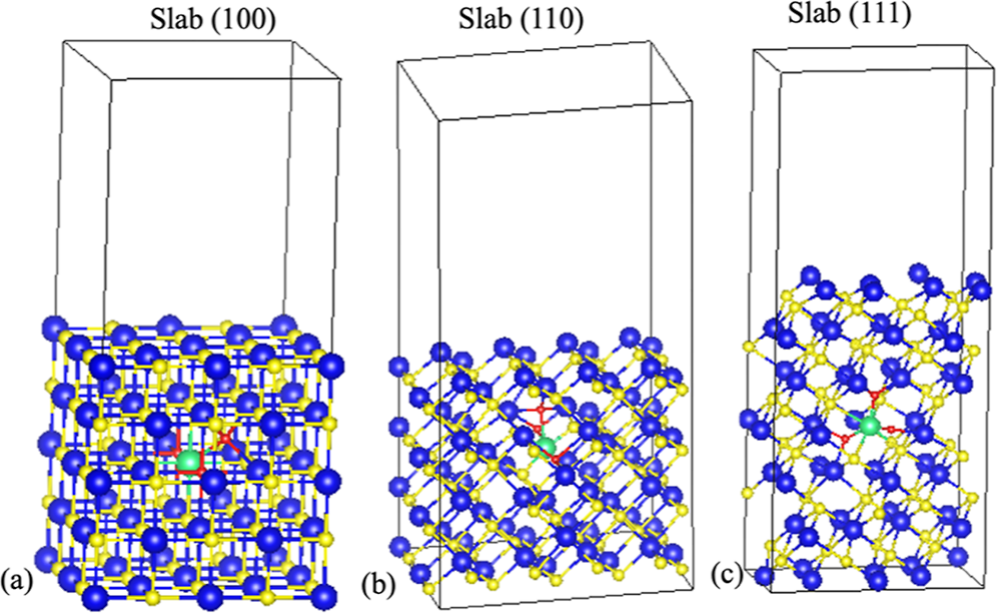
Structural models for PbS slabs in the surface energy calculations with (a) (100), (b) (110), and (c) (111) surfaces. Each slab contains a defect consisting of a Th atom surrounded by two O atoms at substitutional sites and one at an interstitial site. Blue, yellow, green, and red atoms represent lead, sulfur, thorium, and oxygen, respectively.

To resolve the open question of the surface topography change due to the introduction of thorium in PbS films, we calculated the surface energies of PbS (100), (110), and (111) slabs with and without Th incorporation using eq 3 and report them in Table 5.
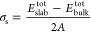
3where *E*_slab_^tot^ is the total energy of the slab, *E*_bulk_^tot^ is the energy of the bulk of the system having the same number of atoms, and *A* is the surface area of the slab. Figure 5 illustrates the slab models used for calculations of surface energies. Sufficiently large values of vacuum spacing (1.5 nm) were used in slab modeling to overcome nonphysical interactions due to the periodic boundary calculations.

**Table 5:** Surface Energy (meV/Ang^2^) of PbS Surfaces Due to the Incorporation of Th and O in PbS^a^

Th_Pb_ + 2*O*_S_ + *I*_O_			
chemical environments	surface energy (meV/Ang^2^)		
	(111)	(100)	(110)
	Pb-terminated		
Pb-rich	–2.8	–23.1	–3.7
S-rich	1.5	–19.3	–1.1
# atoms in slab/model	104	80	128

PbS surface energy (meV/Ang^2^) without thorium and oxygen			
Pb-poor	107.0	11.9	24.3
Pb-rich	68.0		

a The most stable defect configuration is considered for surface energy calculations. The energetically preferred surfaces are underlined.

The surface energy of a solid quantifies the energy required to increase its surface area under zero stress. Consequently, stable solids possess positive surface energy.^34−36^ A negative surface energy suggests that surface formation occurs spontaneously, resulting in the disintegration of the solid and the destabilization of the bulk. Negative surface energies can give rise to the formation of porous equilibrium structures or stable nanoparticles of small size.^35^

We found that the surface energy of the (100) surface is lower than the surface energies of (110) and (111) surfaces for PbS without Th incorporation. Consequently, the (100) surface is preferred and is expected to be the exposed surface in the growth of PbS thin films (without Th incorporation), in agreement with the experimental observations.^23^ In contrast, upon incorporation of Th and O, the (100) and (110) surface energies were negative, suggesting that both surfaces thermodynamically destabilize the bulk of PbS. Only the (111) surface energy in S-rich conditions was found to be positive, enabling bulk crystal growth. Thus the (111) surface is the preferred orientation for the growth of alloyed films in the bulk of PbS. These results are in alignment with the experiments where surface topography was found to change from (100) to (111) due to the introduction of thorium, and they do not appear to require any interaction between Th and GaAs.^23^ Moreover, our results suggest that the alloyed film must be grown under S-rich conditions.

Thus, theoretical modeling of thorium defects in the PbS system revealed unusual properties, such as significant oxygen incorporation, an increased bandgap, and alterations in the surface morphology of the bulk film. The key findings of this work are as follows:

(i) Significant oxygen incorporation was observed in a thorium-doped PbS system, with an average of three oxygen atoms accumulating per Th atom. This result aligns well with experimental findings, where an accumulation of 2.4 oxygen atoms per Th atom was reported.
(ii) From the charge state calculations, we determined that thorium remains stable at a +4 oxidation state within the PbS system, consistent with experimental findings.
(iii) Thorium incorporation into PbS results in an increased band gap, aligning well with experimental observations.
(iv) Surface energy calculations indicate that PbS films preferentially grow in the (111) direction, which is in excellent agreement with experimental results.

## 4 Conclusions

We investigated the unusual defect chemistry of thorium in PbS. We found that thorium accumulates a substantial number of oxygen atoms around it, including one strongly bound adjacent interstitial oxygen. The combined thorium and interstitial oxygen defects manifest a +2 oxidation state in bulk PbS with Th in a +4 state. Doping with Th affects the surface energies, growth morphology, and electronic properties. Notably, an increase in the bandgap was obtained due to the presence of Th in PbS. Finally, we calculated the surface energies of the doped system and found that only (111) surfaces showed positive surface energy. Therefore, growth is preferred in the (111) direction for thorium-incorporated PbS. The computational results are in good agreement with the experimental observations and provide important insights for better understanding them, including oxygen accumulation, crystallographic orientation, and subsequent surface topography change due to Th incorporation in PbS.
